# Molecular and Clinical Epidemiology of SARS-CoV-2 Infection among Vaccinated and Unvaccinated Individuals in a Large Healthcare Organization from New Jersey

**DOI:** 10.3390/v15081699

**Published:** 2023-08-05

**Authors:** José R. Mediavilla, Tara Lozy, Annie Lee, Justine Kim, Veronica W. Kan, Elizabeth Titova, Ashish Amin, Michael C. Zody, André Corvelo, Dayna M. Oschwald, Amy Baldwin, Samantha Fennessey, Jerry M. Zuckerman, Thomas Kirn, Liang Chen, Yanan Zhao, Kar Fai Chow, Tom Maniatis, David S. Perlin, Barry N. Kreiswirth

**Affiliations:** 1 Center for Discovery and Innovation, Hackensack Meridian Health, Nutley, NJ 07110, USA; 2 Department of Pediatrics, Hackensack University Medical Center, Hackensack, NJ 07601, USA; 3 Hackensack Meridian Health Biorepository, Hackensack, NJ 07601, USA; 4 New York Genome Center, New York, NY 10013, USAsfennessey@nygenome.org (S.F.); tmaniatis@nygenome.org (T.M.); 5 Department of Patient Safety and Quality, Hackensack Meridian Health, Edison, NJ 08837, USA; 6 Hackensack Meridian School of Medicine, Nutley, NJ 07110, USA; 7 Public Health and Environmental Laboratories, New Jersey Department of Health, Ewing, NJ 08628, USA; 8 Department of Pathology, Hackensack University Medical Center, Hackensack, NJ 07601, USA

**Keywords:** SARS-CoV-2, COVID-19, pandemic, variants, RT-PCR, sequencing, surveillance, vaccine, breakthrough, comorbidity

## Abstract

New Jersey was among the first states impacted by the COVID-19 pandemic, with one of the highest overall death rates in the nation. Nevertheless, relatively few reports have been published focusing specifically on New Jersey. Here we report on molecular, clinical, and epidemiologic observations, from the largest healthcare network in the state, in a cohort of vaccinated and unvaccinated individuals with laboratory-confirmed SARS-CoV-2 infection. We conducted molecular surveillance of SARS-CoV-2-positive nasopharyngeal swabs collected in nine hospitals from December 2020 through June 2022, using both whole genome sequencing (WGS) and a real-time RT-PCR screening assay targeting spike protein mutations found in variants of concern (VOCs) within our region. De-identified clinical data were obtained retrospectively, including demographics, COVID-19 vaccination status, ICU admission, ventilator support, mortality, and medical history. Statistical analyses were performed to identify associations between SARS-CoV-2 variants, vaccination status, clinical outcomes, and medical risk factors. A total of 5007 SARS-CoV-2-positive nasopharyngeal swabs were successfully screened and/or sequenced. Variant screening identified three predominant VOCs, including Alpha (*n* = 714), Delta (*n* = 1877), and Omicron (*n* = 1802). Omicron isolates were further sub-typed as BA.1 (*n* = 899), BA.2 (*n* = 853), or BA.4/BA.5 (*n* = 50); the remaining 614 isolates were classified as “Other”. Approximately 31.5% (1577/5007) of the samples were associated with vaccine breakthrough infections, which increased in frequency following the emergence of Delta and Omicron. Severe clinical outcomes included ICU admission (336/5007 = 6.7%), ventilator support (236/5007 = 4.7%), and mortality (430/5007 = 8.6%), with increasing age being the most significant contributor to each (*p* < 0.001). Unvaccinated individuals accounted for 79.7% (268/336) of ICU admissions, 78.3% (185/236) of ventilator cases, and 74.4% (320/430) of deaths. Highly significant (*p* < 0.001) increases in mortality were observed in individuals with cardiovascular disease, hypertension, cancer, diabetes, and hyperlipidemia, but not with obesity, thyroid disease, or respiratory disease. Significant differences (*p* < 0.001) in clinical outcomes were also noted between SARS-CoV-2 variants, including Delta, Omicron BA.1, and Omicron BA.2. Vaccination was associated with significantly improved clinical outcomes in our study, despite an increase in breakthrough infections associated with waning immunity, greater antigenic variability, or both. Underlying comorbidities contributed significantly to mortality in both vaccinated and unvaccinated individuals, with increasing risk based on the total number of comorbidities. Real-time RT-PCR-based screening facilitated timely identification of predominant variants using a minimal number of spike protein mutations, with faster turnaround time and reduced cost compared to WGS. Continued evolution of SARS-CoV-2 variants will likely require ongoing surveillance for new VOCs, with real-time assessment of clinical impact.

## 1. Introduction

The ongoing SARS-CoV-2 pandemic which began in December 2019 is arguably the most significant global health episode of the current century [1,2]. In the United States, New Jersey was one of the first states affected and at one point had the highest COVID-19 death rate in the nation [3]. Since the initial case on 1 March 2020, there have been at least 2,575,925 laboratory-confirmed cases in New Jersey, resulting in 172,087 hospitalizations and 36,191 confirmed deaths as of this writing [4]. Our network is currently the largest healthcare organization in the state, with 18 hospitals and more than 500 patient care centers [5]. As of 30 June 2023, we have admitted 46,375 inpatients with a COVID-19 diagnosis and performed over 1,420,580 SARS-CoV-2 tests. Figure S1 depicts the daily number of COVID-19-related hospitalizations in our network since the pandemic began [6], including ICU admissions, ventilator support, and inpatient deaths (see also Figure S2).

The Center for Discovery and Innovation (CDI) is a novel research institute established within our network in January 2019 [7]. In February 2020, the CDI developed the first SARS-CoV-2 real-time reverse-transcription PCR (RT-PCR) assay used by our network for hospital-based COVID-19 testing [8], which was subsequently utilized to perform diagnostic testing from March to October 2020. Archiving SARS-CoV-2 positive samples from hospitals throughout the network facilitated both real-time and retrospective surveillance of SARS-CoV-2 lineages using whole-genome sequencing as well as a previously described assay for rapid detection of specific variants [9]. Weekly surveillance of SARS-CoV-2 positive nasopharyngeal (NP) swabs is still ongoing within the nine largest hospitals.

In December 2020, a large-scale COVID-19 vaccination program was implemented throughout our network. As of this writing, 784,365 individuals have been vaccinated with either the Pfizer-BioNTech (Comirnaty) [10], Moderna (Spikevax) [11], or Janssen (Jcovden) vaccines [12]. From the inception of the program, the CDI acquired and analyzed SARS-CoV-2-positive NP swabs from individuals with a history of COVID-19 vaccination. We previously published on the genomic epidemiology of SARS-CoV-2 breakthrough infections among employees in our organization [13]; however, that report only encompassed the first few months of the vaccination campaign (January–April 2021), prior to the emergence of the Delta and Omicron variants.

Over the course of the pandemic, numerous SARS-CoV-2 lineages have been identified and characterized using genomic surveillance [14,15,16]. In late 2020, distinct variants began to emerge internationally, characterized by specific mutations in the viral spike protein associated with increased binding affinity to the host’s ACE-2 receptor, antibody-mediated immune evasion, or both [17,18,19,20]. Five of these lineages have resulted in widespread transmission, often with increased severity, and have therefore been classified as “variants of concern” (VOCs) [21,22,23]. These include B.1.1.7 (Alpha), B.1.351 (Beta), P.1 (Gamma), B.1.617.2 (Delta), and B.1.5.129 (Omicron), the latter of which has since diversified into successive “waves” of sub-variants and is currently the only lineage classified as a VOC by the U.S. Centers for Disease and Control Prevention (CDC) [24,25]. Other variants with less widespread impact, such as B.1.427/B.1.429 (Epsilon), B.1.526 (Iota), and B.1.621 (Mu), were originally classified as “variants of interest” (VOIs) but have since been re-classified as “variants being monitored” (VBMs), as have Alpha, Beta, Gamma, and Delta.

Numerous studies have investigated the transmissibility and clinical severity associated with particular VOCs, as well as their impact on vaccine efficacy [26,27,28,29,30]. Moreover, underlying comorbidities, including obesity, diabetes, and cardiovascular disease, have been associated with severe clinical outcomes such as hospitalization, ICU admission, and death [31,32,33]. The availability of longitudinal molecular surveillance data from multiple hospitals within our network presents an opportunity to investigate associations between specific SARS-CoV-2 variants, COVID-19 vaccination status, and severe clinical outcomes. In this study, we utilized molecular surveillance data, clinical outcomes, medical risk factors, and vaccination status in order to identify and highlight potential interactions associated with specific SARS-CoV-2 variants among vaccinated and unvaccinated individuals. Overall, the results reinforce the positive impact of COVID-19 vaccination and the public health benefits of conducting genotypic surveillance of SARS-CoV-2 across a large hospital network.

## 2. Materials and Methods

SARS-CoV-2-positive nasopharyngeal (NP) swabs were collected weekly from 9 hospitals across the network, from December 2020 through June 2022. Swabs were delivered to the network biorepository (BioR), de-identified to remove protected health information, and classified by vaccination status [34]. A representative subset of positive swabs were delivered weekly to the CDI for variant screening and genomic surveillance, totaling 10,431 for the study period. Swabs which were successfully screened and/or sequenced (see below) were archived at the CDI and stored at −80 °C. Only swabs which were successfully genotyped, and for which demographic and clinical outcome data were available (see below), were utilized for this study (*n* = 5007).

The viral RNA template was obtained directly from NP swab viral transport medium via treatment with 0.2 mg/mL proteinase-K (Roche Diagnostics, Basel, Switzerland), followed by heat-inactivation at 95 °C for 5 min. The majority of swabs (*n* = 3586) were initially screened via real-time RT-PCR using the SARS-CoV-2 RUO qPCR Primer & Probe Kit (Integrated DNA Technologies, Coralville, IA); the remaining 1421 samples were tested without pre-screening. Swab samples with viral nucleocapsid (N) gene RT-PCR Ct values ≤ 39 were further subjected to variant screening using a previously described assay [9] utilizing the same RNA template. Variant screening assays were performed on a Mic qPCR cycler (Bio Molecular Systems, Upper Coomera, Australia), using a One Step PrimeScript™ RT-PCR Kit (Takara Bio, Shiga, Japan) and custom-designed molecular beacon [35] probes (synthesized at Integrated DNA Technologies or Biosearch Technologies). The original assay was iteratively expanded to target the following spike protein mutations: A67V, ΔH69/V70, L452Q/L452R, T478K, E484A/E484K, F486V, N501Y, and Y505H. Table 1 shows the mutation signatures associated with particular VOCs, as detected by the screening assay.

A subset of successfully screened NP swabs were also subjected to WGS via partnerships with the New York Genome Center (NYGC) and the New Jersey Department of Health Public Health and Environmental Laboratories (NJPHEL); the NYGC methodology was described previously [13,36]. WGS was used to confirm screening assay results, identify specific SARS-CoV-2 lineages and sub-lineages, and distinguish between Beta, Gamma, and Omicron BA.4/BA.5. Genomic sequences were analyzed using Nextstrain [37] and Pangolin [38] and uploaded to GISAID [39]; the SARS-CoV-2 genomes sequenced in this study were deposited in GISAID (https://www.gisaid.org).

Collection of NP swabs associated with vaccine-breakthrough infections began in December 2020, immediately after the start of the vaccination program. Vaccination history was obtained from internal network records, as well as the New Jersey Immunization Information System (NJIIS). Vaccine-related data included information regarding vaccine brand (Pfizer, Moderna, or Janssen) and number/timing of doses. In accordance with CDC criteria [40], subjects were considered “fully vaccinated” if they had received an “initial series” of vaccinations (either 2 doses of Pfizer or Moderna or 1 dose of Janssen). Information regarding additional booster doses was also collected.

De-identified demographic and clinical data were obtained from network health records accessed by the BioR. Available patient data for all 5007 subjects included age, gender, race/ethnicity, and clinical outcome severity (including ICU admission, ventilator usage, and mortality). Risk factor data were available for 3509 subjects and included known history of cancer (including but not limited to bladder, breast, colon, lung, prostate, and skin); cardiovascular disease (including but not limited to arrhythmia, cardiac attack, congestive heart failure, coronary artery disease, and stroke); diabetes; hypertension; hyperlipidemia; obesity; respiratory illness (including but not limited to asthma and chronic obstructive pulmonary disease); and thyroid disease (including but not limited to hypothyroidism). Due to overlap among individual risk factors, a “comorbidity index” was created by combining the overall number of comorbidities (from 0–5) for each individual.

Summary statistics were used for descriptive purposes and included mean with standard deviation, median with interquartile range, or counts with associated frequencies, depending on variable type and underlying distribution. Longitudinal plots, box plots, and bar charts were used to visually assess relationships between variables. Smoothing techniques, such as three-week moving average and log transformations, were applied to the data to assess trends. Correlations between continuous variables were performed using Pearson’s correlation coefficient. Group comparisons were performed using Student’s t-test or Pearson’s chi-squared test, depending on the variable type. A significance level of 0.05 was utilized, and FDR adjustment was used to limit false discoveries due to multiple testing. Multivariate and bivariate logistic regression models using Poisson distribution were used to determine relative risk for vaccine exposure, and odds ratios for all other comparisons. Crude ratios and adjusted ratios for confounding variables identified via the Cochran–Mantel test were reported along with their 95% confidence intervals. Additional exploratory methods included multiple-response testing for repeated measures. All statistical analyses were performed using JMP^®^, Version 17.0. SAS Institute Inc., Cary, NC, USA, 1989–2023.

## 3. Results

### 3.1. SARS-CoV-2 Sample Collection and Selection

**Figure 1:**
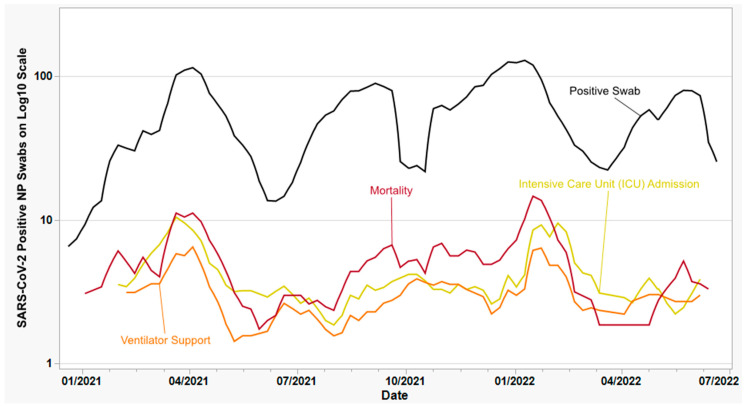
Longitudinal chart depicting the number of SARS-CoV-2-positive nasopharyngeal swabs (black line) selected over the timeframe of the study, juxtaposed with the frequency of severe clinical outcomes within the nine hospitals sampled. Data are shown as a 3-week moving average displayed on a logarithmic scale (*y*-axis). The yellow line represents the weekly number of patients in intensive care (ICU); the orange line represents the weekly number of patients on ventilator support; and the dark red line represents the weekly number of inpatient deaths.

Figure 1 depicts a 3-week moving average of the total number of SARS-CoV-2-positive NP swabs selected for this study, superimposed over the three primary clinical outcomes observed (ICU admission, ventilator support, and mortality). The CDI received a total of 10,431 positive swabs during this period, obtained from 9 hospitals located throughout our network, of which 3261 were related to vaccine-breakthrough infections; the remaining 7170 swabs were classified as “routine”. The majority (*n* = 3586) of positive NP swabs were screened initially using real-time RT-PCR, and only specimens with a Ct value ≤ 39 were selected for further analysis using the variant screening assay; the remaining samples (*n* = 1421) were not pre-screened. For the purposes of this study, only successfully screened swabs with clinical outcome data were included in the final dataset, corresponding to 3430 routine swabs and 1577 vaccine-breakthrough swabs (total = 5007).

### 3.2. Variant Screening of SARS-CoV-2-Positive Nasopharyngeal Swabs

Beginning in December 2020, de-identified SARS-CoV-2-positive NP swabs were screened weekly using a molecular beacon-based real-time PCR assay via melting curve analysis [9]. Through May 2021, only mutations in the E484 and N501 spike protein residues were targeted, as these were sufficient to identify the Alpha variant (N501Y) and partially identify the Beta or Gamma variants (co-occurrence of E484K and N501Y). In this study, 714 swabs were found to harbor the Alpha variant during the same period vs. only 54 putative Beta/Gamma variants (subsequent WGS determined that 39 = Gamma, 14 = Mu, and 1 = Beta). Following the emergence of the Delta variant in May 2022, two additional targets (L452R and T478K) were added to the screening assay, with subsequent identification of 1877 swabs harboring the Delta variant.

The emergence of multiple Omicron variants beginning in December 2021 allowed us to repurpose the E484 and N501 probes, since all Omicron strains harbor E484A and N501Y. The ΔH69/V70 deletion target (present in BA.1), responsible for the “S-gene dropout” in certain diagnostic tests [41,42], was added in February 2022 to distinguish Omicron BA.1 from BA.2. From March 2022 to June 2022, there was a BA.2 wave in the United States caused by the Omicron sub-variant BA.2.12.1, readily identified via detection of a characteristic L452Q mutation, using the aforementioned L452 probe. Beginning in May 2022, co-occurrence of L452R with the ΔH69/V70 deletion further allowed us to infer the presence of BA.4 and/or BA.5 lineages (but not to distinguish between them). Using this combination of targets, we were able to identify 1802 Omicron variants through the end of June 2022, including 899 BA.1, 853 BA.2 (including 469 BA.2.12.1), and 50 BA.4/BA.5 sub-variants. The continued proliferation of novel BA.2 and BA.5 sub-lineages has necessitated incorporation of additional spike gene targets, including, but not limited to, R346T, N440K, K444T, and V445P.

### 3.3. Whole Genome Sequencing of SARS-CoV-2-Positive Nasopharyngeal Swabs

WGS was also performed on a subset of successfully screened isolates (*n* = 3023). Among these, WGS confirmed the following VOCs: 251 Alpha (B.1.1.7), 1 Beta (B.1.351), 39 Gamma (P.1), 693 Delta (B.1.617.2 and sub-lineages), and 1456 Omicron (B.1.1.529 and sub-lineages). Among the Delta sequences, B.1.617.2 (*n* = 187) was the most prevalent lineage; however, numerous sub-lineages were also identified, most notably AY.3 (*n* = 66), AY.4 (*n* = 21), AY.25 (*n* = 86), AY.39 (*n* = 33), AY.44 (*n* = 50), AY.100 (*n* = 25), AY.103 (*n* = 97), and AY.119 (*n* = 25). Among the Omicron sequences, the following lineages were identified through the end of June 2022: B.1.1.529.1 (*n* = 682), consisting primarily of BA.1 (*n* = 204) and BA.1.1 (*n* = 452); B.1.1.529.2 (*n* = 722), consisting primarily of BA.2 (*n* = 191), BA.2.12.1 (*n* = 397), BA.2.3 (*n* = 39), and BA.2.9 (*n* = 55); and several BA.4 (*n* = 16) and BA.5 (*n* = 26)- related lineages. A small number of Omicron BA.1/BA.2 recombinants were also identified, including XN (*n* = 1), XQ (*n* = 5), XT (*n* = 1), and XZ (*n* = 1); all of these were previously classified as BA.2 by our screening assay.

Several VOIs which could not be definitively identified via the screening assay were also identified via WGS, most notably 234 Iota (B.1.526), a predominant lineage in the New York/New Jersey region during the same time period as Alpha, as well as 15 Mu (B.1.621), 9 Epsilon (B.1.427/B.1.429), 1 Kappa (B.1.617.1), 1 Lambda (C.37), and 1 Zeta (P.2). A number of “pre-variant” lineages were also identified from December 2020 to March 2021, including: B.1 (*n* = 21), B.1.1.434 (*n* = 28), B.1.1.519 (*n* = 12), B.1.2 (*n* = 70), B.1.234 (*n* = 10), B.1.243 (*n* = 21), B.1.311 (*n* = 8), B.1.409 (*n* = 8), B.1.575 (*n* = 32), B.1.596 (*n* = 11), B.1.628 (*n* = 8), B.1.637 (*n* = 14), C.2 (*n* = 7), and R.1 (*n* = 10). The R.1 variant was notably associated with a large outbreak at a behavioral health facility within our network in November 2020 [36]. For the purposes of this study, the aforementioned VOIs were classified as “Other”.

**Figure 2:**
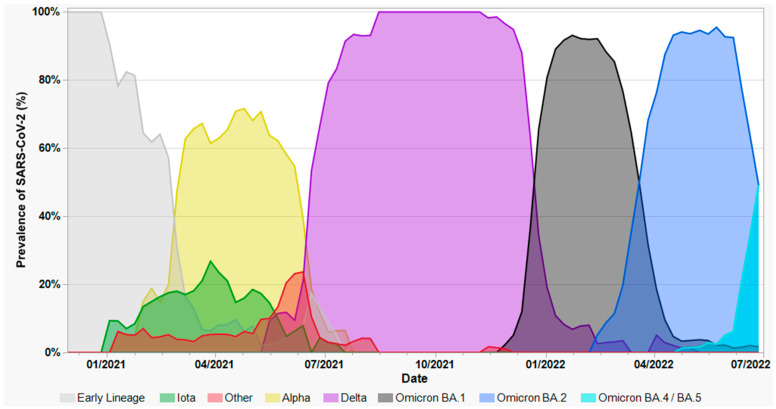
Prevalence of SARS-CoV-2 lineages, VOCs, and VOIs identified in this study. From left to right, gray denotes pre-variant lineages, including B.1, B.1.1.434, B.1.1.519, B.1.2, B.1.234, B.1.243, B.1.311, B.1.409, B.1.575, B.1.596, B.1.628, B.1.637, C.2, and R.1; green denotes Iota (B.1.526); yellow denotes Alpha (B.1.1.7); red denotes other VOCs/VOIs including Beta (B.1.351), Gamma (P.1), Epsilon (B.1.427/1.429), Kappa (B.1.617.1), Lambda (C.37), Mu (B.1.621), and Zeta (P.2); purple denotes Delta (B.1.617.1) and its sub-lineages, including AY.3, AY.4, AY.25, AY.39, AY.44, AY.100, AY.103, and AY.119; black denotes Omicron BA.1, BA.1.1, and related sub-lineages; blue denotes Omicron BA.2 and related sub-lineages, including BA.2.12.1; and turquoise denotes the initial emergence of BA.4 and BA.5 lineages.

Figure 2 depicts the chronological succession of variant “waves” observed in this study, derived from the combined results of variant screening and WGS. Multiple pre-variant lineages circulated during 2020 and the first few months of 2021, with Iota and Alpha both emerging in January 2021. The latter two predominated for the first half of 2021, punctuated by a handful of other variants, including Gamma and Mu, but were displaced by the emergence of Delta in May 2021. From August to November 2021, Delta sub-lineages accounted for nearly 100% of all swabs, only to be displaced beginning in December 2021 by successive waves of Omicron sub-lineages. Although the timeframe encompassed by this study only extends through June 2022, this dynamic continues, characterized primarily by sub-variants of BA.5 (e.g., BQ.1.1) and BA.2 (e.g., XBB.1.5).

### 3.4. COVID-19 Vaccination Status and Vaccine-Breakthrough Infections

Vaccination against SARS-CoV-2 was undertaken by our network beginning in December 2020. Among the 5007 de-identified subjects with SARS-CoV-2 infection represented in this study, 1577 (31.5%) had a history of vaccination, either with the Pfizer-BioNTech (*n* = 812, 51.5%), Moderna (*n* = 616, 39.1%), or Janssen (*n* = 121, 7.7%) vaccines or a combination thereof (*n* = 28, 1.8%). Among the 1577 vaccinated individuals, 967 (61.3%) only received an initial vaccine series, defined as 2 doses of Pfizer and/or Moderna or 1 dose of Janssen; only 400 (25.4%) went on to receive additional booster doses. The median number of days from the initial vaccine dose to a positive SARS-CoV-2 test result was 275 days (IQR 184–365), while the median number of days since the most recent vaccine dose was 181 days (IQR 108–254).

**Figure 3:**
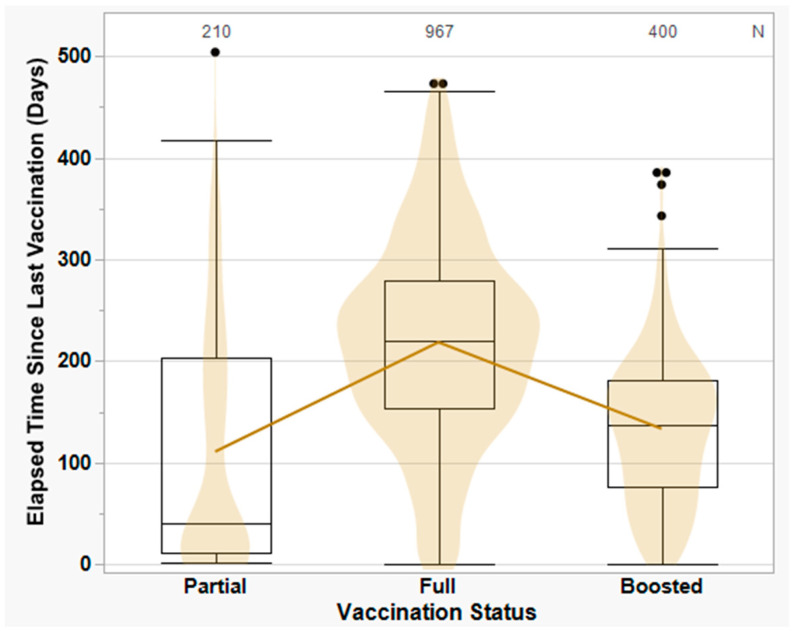
Number of days to positive SARS-CoV-2 diagnosis since the last COVID-19 vaccine dose received, stratified by number of vaccine doses (regardless of vaccine brand). Numbers at the top of the figure denote the number of study subjects receiving a particular number of vaccine doses; black points represent outliers. “Partial” refers to a single dose of either Pfizer (*n* = 116) or Moderna (*n* = 94) mRNA vaccine; “full” refers to two doses of Pfizer (*n* = 498) or Moderna (*n* = 348) or one dose of Janssen (*n* = 121); and “boosted” refers to any additional doses of Pfizer (*n* = 198), Moderna (*n* = 174), or both (*n* = 28). Trendline denotes the mean number of days to positive SARS-CoV-2 diagnosis, while horizontal lines inside the boxplots denote the median number of days (41 days partially vaccinated, 220 for fully vaccinated, and 138 for boosted).

For simplicity, vaccine doses were stratified as follows: (a) partially vaccinated, defined as a single dose of either Moderna or Pfizer (median = 41 days, IQR 11–204); (b) fully vaccinated, defined as two doses of either Moderna or Pfizer or a single dose of Janssen (median = 220 days, IQR 153–279); and (c) boosted, defined as any additional doses of Moderna, Pfizer, or both (median = 138 days, IQR 77–181) (Figure 3). We looked more closely at “full” vaccination to see if there were any brand-related differences between 2 doses of Pfizer (median = 211 days, IQR 150–274), 2 doses of Moderna (median = 235 days, IQR 174–300), or 1 dose of Janssen (median = 189 days, IQR 135–178); the differences were not significant, however, and were limited to the first few months of the vaccination campaign.

Table S1 describes the demographic characteristics of the 5007 de-identified subjects represented in this study, including age, race/ethnicity, and gender, stratified further by COVID-19 vaccination status. Vaccination was positively correlated with age (Pearson’s correlation = 0.22, *p* < 0.001), regardless of other variables, with highly significant differences observed among younger and older age groups. The median age for the overall study was 55 (IQR 34–72), with differences observed for males vs. females (58 vs. 54, respectively); vaccinated vs. unvaccinated individuals (63 vs. 51); vaccinated vs. unvaccinated males (68 vs. 52); vaccinated vs. unvaccinated females (58 vs. 50); and vaccinated males vs. vaccinated females (68 vs. 58). Overall, 56.4% of females were vaccinated vs. only 43.6% of males, with males being 16% less likely to be vaccinated than females (RR_CRUDE_ = 0.84, 95% CI [0.73, 0.96], *p* = 0.01).

Vaccination rates were highest among individuals identifying as Asian (65/171 = 38.0%), followed by White (1039/2906 = 35.7%), Hispanic (203/907 = 22.4%), and Black (113/509 = 22.2%). A significantly higher proportion of individuals who identified as White were vaccinated (*p* < 0.001); by contrast, a significantly higher proportion of individuals who identified as Hispanic or Black/African-American were unvaccinated (*p* < 0.001). Despite these disparities, vaccinated Black and Hispanic individuals were 49% (OR = 0.51, 95% CI [0.41, 0.64], *p* < 0.001) and 48% (OR = 0.52, 95% CI [0.44, 0.62] *p* < 0.001) less likely to test positive for SARS-CoV-2 than White individuals, respectively. However, this may reflect lower vaccination rates in these populations, rather than a protective effect.

**Figure 4:**
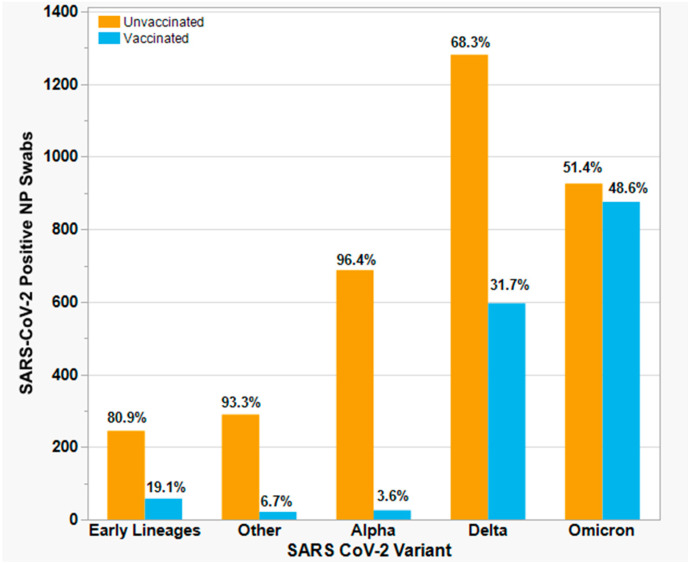
Relative frequency of infection by SARS-CoV-2 variants among vaccinated and unvaccinated individuals. Variants are arranged in chronological order of emergence within the New Jersey region. “Vaccinated” is defined as a known history of vaccination in individuals with laboratory-confirmed SARS-CoV-2 infection (i.e., vaccine-breakthrough infection). “Other” denotes VOCs/VOIs circulating concurrently with Alpha in early 2021; “Delta” includes B.1.617.2 and associated sub-lineages; “Omicron” includes BA.1, BA.2, and BA.4/BA.5.

No significant differences were observed among variants or sub-variants infecting vaccinated vs. unvaccinated individuals, with the exception of Alpha (*p* < 0.001) and pre-variant lineages (*p* < 0.001), both of which coincided with the first few months of the vaccination campaign (Table S1). Vaccinated individuals accounted for a higher proportion of individuals infected with Omicron BA.1 and BA.2 variants, but this difference was not significant. A notable increase in vaccine-breakthrough swabs was observed in August 2021, continuing during the periods of Delta and Omicron predominance (despite an increasing likelihood of being vaccinated over time). During the Omicron period, 48.6% (876/1802) of all positive NP swabs collected by the BioR corresponded to vaccine-breakthrough infections, consistent with the sample distribution in this study (Figure 4). SARS-CoV-2 variants associated with vaccine breakthroughs generally reflected predominant variants in circulation during a given time period, as reported previously [13].

### 3.5. Severe Clinical Outcomes among Vaccinated vs. Unvaccinated Individuals

Three primary outcome measures were analyzed, including admission to an intensive care unit (336/5007 = 6.7%), ventilator support (236/5007 = 4.7%), and mortality (430/5007 = 8.6%). Table 2 summarizes data for all three outcomes, stratified by vaccination and demographics. Significant differences were found among age and gender for each clinical outcome, and to a lesser extent for some racial groups. After controlling for sociodemographic factors, however, significant differences were only observed for age, with gender also being significant for mortality.

**Figure 5:**
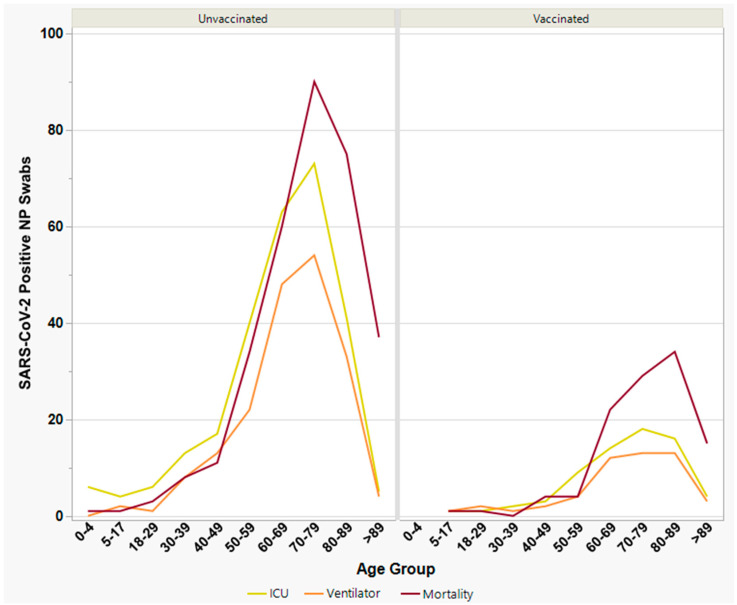
Number of severe clinical outcomes by age group, among unvaccinated (**left panel**) vs. vaccinated (**right panel**) individuals with laboratory-confirmed SARS-CoV-2 infection. The yellow line denotes ICU admission; the orange line denotes ventilator supports; and the dark red line denotes mortality. Median ages for ICU admission, ventilator usage, and mortality were 67, 69, and 74, respectively, for unvaccinated individuals; and 72, 72, and 79, respectively, for vaccinated individuals.

Figure 5 depicts the frequency of severe clinical outcomes by age group among vaccinated vs. unvaccinated individuals, depicting a shift towards higher median ages for vaccinated individuals. Additional outcome-specific factors are discussed separately in the following three sections.

#### 3.5.1. Intensive Care Unit (ICU) Admission Individuals

Among the 336 individuals admitted to an ICU, 197 (58.6%) and 139 (41.4%) were male and female, respectively. Males were 1.71 times likelier to be admitted to the ICU (OR = 1.71, 95% CI [1.37, 2.14], *p* < 0.001) regardless of vaccination status, accounting for 57.5% (154/268) of unvaccinated ICU admissions and 63.2% (43/68) of vaccinated admissions. Among males admitted to the ICU, 78.2% (154/197) were unvaccinated and 21.8% (43/197) were vaccinated, while among females, 82.0% (114/139) were unvaccinated and 18.0% (25/139) were vaccinated.

The median age of individuals admitted to the ICU was 68 (IQR 57–78), while that of individuals who were not admitted was 54 (IQR 32–71). The vast majority (84.2%) of admissions were among individuals > 50 years of age, with nearly a third (91/283 = 32.2%) occurring among individuals between 70 and 79 years old. Overall, the risk of ICU admission was 1.8 times higher among unvaccinated individuals (RR_CRUDE_ = 1.81, 95% CI [1.40, 2.35], *p* < 0.001), representing 7.8% (268/3430) of unvaccinated individuals vs. 4.3% (68/1577) of vaccinated individuals. The overall mortality rate was 69.0% (232/336) among those admitted to the ICU, with unvaccinated patients accounting for 82.3% (191/232) of deaths; this difference was not significant, however (RR_CRUDE_ = 1.18, 95% CI [0.96, 1.45], *p* = 0.11).

Significant differences in ICU admission rates were not observed between different vaccine brands, whether overall among all vaccinated individuals (*p* = 0.44), or among those only receiving an initial vaccination series (*p* = 0.26). Nevertheless, among fully vaccinated individuals in the ICU, a 14% increase in individuals that had received Moderna (*n* = 21) compared to Pfizer (*n* = 18) was observed, despite a higher frequency of Pfizer vaccine uptake among study subjects overall.

#### 3.5.2. Ventilator Support

A total of 236 individuals in this study were placed on a ventilator, the vast majority (218/236 = 92.4%) of which were also admitted to an ICU. A Cochran–Mantel test was performed to account for ICU admission as a potential confounding variable, but the results were not significant (*p* = 0.30). Demographics for individuals requiring ventilation were similar to those for ICU admission, with a median age of 70 (IQR 60–79), and comprised 59.7% males (141/236) and 40.3% females (95/236). Males were 1.77 times likelier to need a ventilator compared to females (OR = 1.77, 95% CI [1.36, 2.32], *p* < 0.001) and comprised a higher proportion of patients on ventilators regardless of vaccination status.

Unvaccinated individuals were 1.85 times likelier to need ventilator support (RR_CRUDE_ = 1.85, 95% CI [1.30, 2.69], *p* < 0.001), corresponding to 78.4% (185/236) of unvaccinated patients vs. 21.6% (51/236) of vaccinated patients. No significant differences were observed between vaccine brands, whether among all vaccinated individuals (*p* = 0.46), or among those only receiving an initial series (*p* = 0.24). Similarly to fully vaccinated individuals in the ICU, a higher number of patients on ventilators received Moderna (*n* = 20) compared to Pfizer (*n* = 16). The overall mortality rate was 76.7% (181/236) among patients placed on ventilators, with unvaccinated patients accounting for 85.1% (154/181) of deaths.

#### 3.5.3. Mortality

Mortality was the third clinical outcome we investigated, with an overall mortality rate of 8.6% (430/5007) noted in this study. Of the 430 individuals who died, 232 (53.9%) were admitted to the ICU, and 181 (42.1%) were placed on ventilators. Demographics were similar to those for the other two outcomes, with median ages of 53 (IQR 32–70) and 75 (IQR 65–85) for survivors and deceased individuals, respectively, and similar proportions for males (245/430 = 57.0%) and females (185/430 = 43.0%). Overall, males were 1.61 times likelier to die than females (OR = 1.61, 95% CI [1.31, 1.96], *p* < 0.001). Whites accounted for the majority of deceased individuals (304/430 = 70.7%), followed by individuals identifying as Hispanic (58/430 = 13.5%), Black (25/430 = 5.8%), and Asian (16/430 = 3.7%). Overall, 10.5% of Whites died, followed by 9.4%, 6.4%, and 4.9% of individuals identifying as Asian, Hispanic, and Black, respectively.

Among the 430 individuals who died, 110 (25.6%) were vaccinated and 320 (74.4%) were unvaccinated (*p* < 0.001), representing an overall mortality rate of 7.0% (110/1577) and 9.3% (320/3430), respectively. Unvaccinated individuals were 1.4 times likelier to die (RR = 1.44, 95% CI [1.11, 1.87], *p* = 0.01). Median ages at time of death were 75 (IQR 63–85) and 79 (IQR 68–87) for unvaccinated and vaccinated individuals, respectively, with 90.1% (100/110) of deaths among vaccinated individuals occurring in people ≥ 60 years old, compared to 81.9% (262/320) of deaths among unvaccinated individuals.

In contrast to ICU admission and ventilator support, significant differences were observed for mortality among different vaccine brands (*p* = 0.03), with a greater proportion of deaths seen among individuals vaccinated with Janssen (*n* = 14/121, 11.6%) compared to Moderna (*n* = 50/616, 8.1%) or Pfizer (*n* = 46/812, 5.7%). After further stratification by vaccine dose and SARS-CoV-2 variant, the odds of death were lowest for Pfizer compared to Moderna or Janssen (initial vaccine series: Pfizer vs. Moderna OR = 0.59, 95% CI [0.36, 0.95], *p* = 0.03; Pfizer vs. Janssen OR = 0.43, 95% CI [0.22, 0.84], *p* = 0.01). However, these differences were largely attributable to the Delta variant (Pfizer vs. Moderna OR = 0.39, 95% CI [0.17, 0.93], *p* = 0.04; Pfizer vs. Janssen OR = 0.46, 95% CI [0.25, 0.86], *p* = 0.01). By contrast, no significant differences between vaccine brands were observed during the Omicron period (*p* = 0.14), although a similar trend in mortality was observed.

### 3.6. Risk Factors and Comorbidities among Vaccinated vs. Unvaccinated Individuals

In addition to clinical outcomes, medical history was available for 3509/5007 (70.0%) of the patients in this study, encompassing risk factors including but not limited to: cardiovascular disease (1872/3509 = 53.3%), hypertension (1738/3509 = 49.5%), hyperlipidemia (1199/3509 = 34.2%), diabetes (799/3509 = 22.8%), respiratory illness (687/3509 = 19.6%), cancer (455/3509 = 13.0%), thyroid disease (442/3509 = 12.6%), and obesity (171/3509 = 4.9%). Table 3 shows highly significant (*p* < 0.001) contributions to mortality by cardiovascular disease, hypertension, cancer, diabetes, and hyperlipidemia; by contrast, obesity, respiratory disease, and thyroid disease were not significantly associated with mortality.

**Figure 6:**
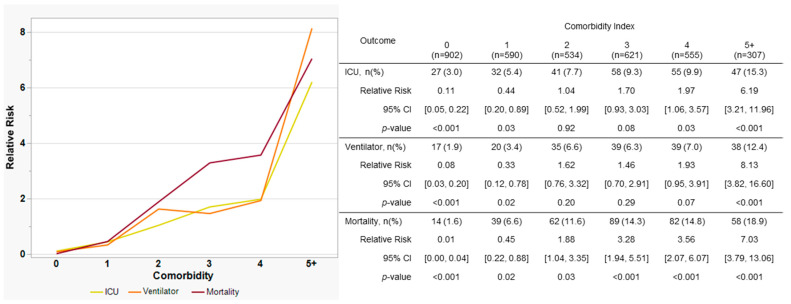
Relationship between total number of comorbidities (comorbidity index) and risk of severe clinical outcome. Yellow line, ICU admission; orange line, ventilator usage; and dark red line, mortality. Comorbidities included cancer, cardiovascular disease, diabetes, hypertension, hyperlipidemia, obesity, respiratory illness, and thyroid disease. Individuals with ≥5 comorbidities were combined due to small sample size. CI, confidence interval; ICU, intensive care unit.

Considerable overlap among risk factors was observed, particularly between cardiovascular disease and hypertension, but also between diabetes and hyperlipidemia. We therefore re-classified distinct risk factors as comorbidities and investigated whether the overall number of comorbidities, or “comorbidity index”, was significantly associated with severe outcomes. Figure 6 depicts the relationship between increased comorbidity index and risk of severe clinical outcome (ICU admission, ventilator support, or death). An increase in risk was observed for all three outcomes as the comorbidity index increased, especially for mortality. Individuals with no comorbidities were 89%, 92%, and 99% less likely to be admitted into the ICU, placed on a ventilator, or die, respectively. By contrast, individuals with five or more comorbidities were 6.2, 8.1, and 7.0 times likelier to be admitted, require ventilator therapy, or die, respectively.

### 3.7. Analysis of SARS-CoV-2 Variants and Severe Clinical

In addition to medical risk factors, we also sought to understand whether infection with distinct SARS-CoV-2 variants led to differences in severe clinical outcomes. For the purpose of this analysis, the following variant classifications were used: “Early Lineage” (2020 through early 2021), “Other” (VOCs and VOIs other than Alpha from early 2021 onward), Alpha (B.1.1.7), Delta (B.1.617.2 and sub-lineages), Omicron BA.1 (B.1.1.529.1 and sub-lineages), Omicron BA.2 (B.1.1.529.2 and sub-lineages), and Omicron BA.4/BA.5 (B.1.1.529.4, B.1.1.529.5, and their respective sub-lineages). Overall, variant type was highly significant (*p* < 0.001) in simple bivariate analyses for all three clinical outcomes when stratified by vaccination status, including ICU admission, ventilator support, and mortality (Table S2).

A generalized linear regression model was then used to look at inter-variant differences more closely. Significant differences were observed for all three clinical outcomes, driven primarily by Delta and Omicron BA.1 for ICU admission and ventilator support, and Omicron BA.1 for mortality. The proportion of vaccinated individuals experiencing severe clinical outcomes was generally lower than that of unvaccinated individuals, except for Alpha, “Other”, and Omicron BA.4/BA.5; however, the latter involved low numbers of vaccinated individuals.

Age-related differences were also observed between different variant types and severe outcomes. Median ages were higher among vaccinated individuals with Delta infection for all three outcomes, including ICU admission (73 vaccinated vs. 66 unvaccinated), ventilator support (72 vs. 69), and mortality (78 vs. 74). By contrast, median ages for individuals with Omicron infection were similar for vaccinated and unvaccinated individuals, including ICU admission (69 vs. 69), ventilator support (68 vs. 71), and mortality (77 vs. 75). After stratifying Omicron into BA.1 vs. BA.2, however, marked differences were observed. Among vaccinated individuals with BA.1 infection, median ages were lower for ICU admission (66 vs. 69) and ventilator support (66 vs. 70), but higher for mortality (79 vs. 72). By contrast, median ages were lower among vaccinated individuals with BA.2 infection, including ICU admission (71 vs. 77), ventilator support (71 vs. 78), and mortality (73 vs. 87).

**Figure 7:**
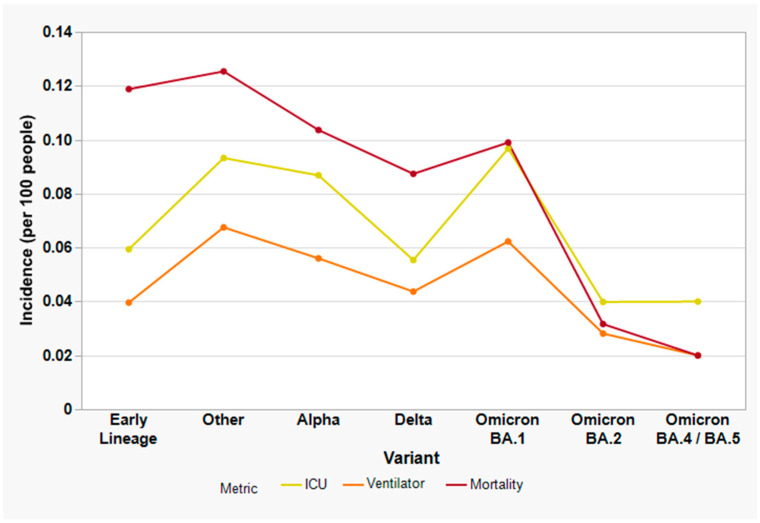
Incidence of severe clinical outcomes among 5007 study subjects, stratified by SARS-CoV-2 variants displayed in chronological order of emergence. “Other” refers to variants of concern/interest other than Alpha which were circulating in early 2021 (e.g., Iota, Gamma, Mu, etc.). Yellow line, ICU admission; orange line, ventilator support; dark red line, mortality.

We further ranked the variants by their relative frequencies among each of the clinical outcomes (Figure 7). An overall downward trend in mortality and ventilator usage was observed over time (*p* < 0.001), corresponding to the temporal progression of distinct variants. However, the Omicron BA.1 wave resulted in a significant increase in all three outcomes, followed by markedly reduced incidences during the BA.2 wave. Overall, infection with earlier variants classified as “Other” was 4.36 times more deadly than the other variants (RR_CRUDE_ = 4.36, 95% CI [1.69, 13.68], *p* < 0.001) followed by Alpha with a RR_CRUDE_ = 2.72 (95% CI [1.23, 7.85], *p* = 0.02) and Omicron BA.1 with a RR_CRUDE_ = 2.15 (95% CI [1.01, 6.13], *p* = 0.07). There was an 89% reduction in mortality during the period when Omicron BA.2 was dominant (RR_CRUDE_ = 0.11, 95% CI [0.04, 0.35], *p* < 0.001]).

Unvaccinated individuals with BA.1 infection were 4.8 times as likely to be admitted to the ICU (RR_ADJUSTED_ = 4.83, 95% CI [2.73, 8.85], *p* < 0.001); compared to unvaccinated individuals with BA.2 infection who were less than half as likely to be admitted (RR_ADJUSTED_ = 2.06, 95% CI [0.92, 4.66], *p* = 0.08), a difference which was not significant. Unvaccinated individuals with BA.1 infection were even likelier to require ventilator support (RR_ADJUSTED_ = 7.68, 95% CI [3.63, 17.87], *p* < 0.001), which was almost 5 times higher compared to unvaccinated individuals with BA.2 infection (RR_ADJUSTED_ = 1.54, 95% CI [0.58, 4.01], *p* = 0.37); this difference was also not significant. Lastly, unvaccinated individuals with BA.1 infection were more than three times as likely to die as vaccinated individuals (RR_ADJUSTED_ = 3.38, 95% CI [1.95, 5.96], *p* < 0.001), representing a 44% increase compared to unvaccinated individuals with BA.2 infection (RR_ADJUSTED_ = 2.35, 95% CI [0.94, 5.91], *p* = 0.07); however, this difference was also not significant (Table S2).

Notably, significant differences in mortality were observed between different vaccine brands during the period when Delta was predominant, with deaths observed among 6.3% (*n* = 20), 12.6% (*n* = 25), and 14.8% (*n* = 9) of individuals vaccinated with Pfizer, Moderna, and Janssen, respectively. Vaccination with Pfizer appeared to offer the most protection against Delta-associated mortality, exhibiting a 61% lower risk of death compared to Moderna (OR = 0.39, 95% CI [0.17, 0.93], *p* = 0.04), and a 54% lower risk compared to Janssen (OR = 0.46, 95% CI [0.25, 0.86], *p* = 0.01). However, no significant differences were found between vaccine brands among the other variants in this study, including Omicron.

## 4. Discussion

The SARS-CoV-2 pandemic which began in late 2019 is noteworthy among recent pandemics thanks to the unprecedented scope of genomic surveillance [43,44], as well as the rapid development of multiple vaccines [43,44]. The initial promise displayed by novel COVID-19 vaccines [45] was subsequently tempered by the emergence of SARS-CoV-2 variants with increasingly diverse spike protein mutations, resulting in greater transmissibility and evasion of antibody-mediated immunity [19,46,47]. Despite this ongoing challenge, however, numerous studies continue to highlight significant reductions in morbidity and mortality as a result of vaccination [30,48,49,50,51,52,53,54].

As of June 2023, the United States remains among the nations most impacted by the pandemic, with the largest official count of cases (107,303,873) and deaths (1,168,100), and the fourth highest death rate among OECD nations [55]. Within the United States, New Jersey was one of the first states affected and also one of the worst [55,56]. During the first year of the pandemic, it ranked among the states with the highest COVID-19 hospitalization and mortality rates and in December 2020, had the highest death rate in the world [57]. A number of peer-reviewed studies have addressed various aspects of the pandemic in New Jersey [8,31,58,59,60,61], but few are comprehensive, especially with regard to molecular surveillance and vaccination.

The trajectory of the pandemic within our healthcare network, as shown in Figure S1, closely parallels that of New Jersey overall [62]. There were three major surges in hospitalization and other severe outcomes, corresponding roughly to the initial outbreak in early 2020; the winter of 2020–2021; and the winter of 2021–2022. Following the implementation of vaccines against COVID-19 in December 2020, a decline in hospitalizations and severe outcomes was observed. Although the Delta wave (May–November 2021) was particularly severe in many regions of the United States, it was associated with relatively few hospitalizations and deaths in our network. By contrast, the first Omicron surge (December 2021–February 2022) was associated with the highest level of hospitalization since the start of the pandemic. Severe outcomes tracked closely with hospitalization until the initial Omicron BA.1 surge, following which they have become increasingly decoupled, with no subsequent spikes or waves.

Our network has been conducting weekly variant surveillance since December 2020 using the aforementioned real-time RT-PCR-based screening assay [9]. In this study, we focused on an 18-month period ending in June 2022, which allowed us to fully capture the first two Omicron waves (BA.1 and BA.2). A salient feature of our assay has been the ability to robustly identify geographically relevant VOCs and sub-variants using a minimal set of spike gene mutation targets (L452Q/R, T478K, E484A/K, N501Y, and ΔH69/V70). This approach was sufficient throughout the period encompassed by this study, which was dominated by VOCs (Alpha, Delta, and Omicron) readily characterized by a combination of these five targets (Table 1). Although the aforementioned mutations could not discriminate between Beta and Gamma, these were less prevalent in our region, and the majority were subsequently confirmed as Gamma via WGS. Since June 2022, the proliferation of BA.2- and BA.5-related sub-variants has necessitated the incorporation of additional targets, including R346T, N440K, K444T, and V445P [63].

PCR-based surveillance approaches allow for timely identification of predominant and emergent viral lineages, without the cost and labor associated with WGS. Even with availability of in-house sequencing, turnaround times for WGS may entail several days, or even longer for large numbers of samples. By contrast, PCR-based screening assays such as ours [9] can deliver informative results within a single day, without the need for library preparation, costly materials, or expensive instrumentation [64,65,66]. Moreover, screening data can also inform clinical decisions regarding the use of monoclonal antibodies, many of which have been rendered ineffective by successive accumulation of particular spike gene mutations [63,67,68,69,70]. Various real-time PCR-based screening assays capable of detecting multiple VOCs have been described [64,66,71,72,73], but to our knowledge have not been utilized as extensively, except perhaps for the one described by Moisan et al., which was used to analyze 2221 patient samples [74].

In this study, we employed a retrospective cohort design involving SARS-CoV-2 positive swab specimens from individuals with known vaccination history, allowing us to investigate potential interactions between vaccination status, demographics, medical risk factors, and SARS-CoV-2 variants. Various studies have addressed one or more of these aspects, but not comprehensively [26,32,33,75]. Vaccine uptake was strongly correlated with age in our study, especially among the 5–18 and > 70 year old age groups; however, pediatric vaccinations for children ≤ 5 years of age were not approved until 18 June 2022 [76], so our analysis was not extended to this age group. Vaccine-breakthrough infections were more common among older age groups, despite higher levels of vaccination. Gender was also significant, with males being less likely to be vaccinated than females; this finding is in agreement with the U.S. Census Bureau Household Pulse Survey [77], although other studies suggest men may be likelier to be vaccinated [78,79].

During the first half of 2021, the number of vaccine-breakthrough swabs received was low, potentially explained by increasing numbers of fully vaccinated individuals in our population, as well as close antigenic matches between circulating variants and the original Wuhan-Hu-1 vaccine strain [80]. Breakthrough infections during this period typically occurred in partially or recently vaccinated individuals. The number of vaccine-breakthroughs increased halfway through the Delta period (August 2021), and rose sharply after the emergence of Omicron in December 2021, following which nearly half of all positive swabs collected were breakthrough-related (Figure 4). Notably, Figure 3 suggests there may have been a stronger protective effect for two doses (full vaccination) compared to additional booster doses. However, since most breakthrough infections in individuals receiving booster doses occurred during the Omicron period, this finding may be explained by increasingly poor matches between spike protein sequences from emerging variants and the original vaccine strain [81,82], as well as by fewer individuals receiving additional booster doses over time [34].

A primary goal of our study was to evaluate the effect of different SARS-CoV-2 variants on severe clinical outcomes among vaccinated vs. unvaccinated individuals. We did not however investigate overall hospitalization, partly due to issues in distinguishing patients hospitalized “for COVID” vs. “with COVID” [83]. Unvaccinated individuals were invariably at higher risk for experiencing severe outcomes, including ICU admission, ventilator support, and mortality. Notably, these estimates do not include severe outcomes from early 2020, prior to the availability of vaccination. Age was the single most significant predictor of severe clinical outcome in our study, with gender being significant only for mortality. Vaccination tended to shift median ages for all three outcomes towards older subgroups (Figure 5). Males experienced worse outcomes than females regardless of vaccination status, which may be due to biological factors [84,85,86,87,88], in addition to behavioral differences [77,89]. Significant differences were observed between vaccine brands, but only for mortality, with Pfizer appearing to be most protective, followed by Moderna, then Janssen (which is no longer available in the U.S. as of May 2023 [90]).

Underlying comorbidities have been implicated repeatedly in poor clinical outcomes among individuals with COVID-19 disease [31,33,91]. Surprisingly, individual comorbidities were not significant in simple bivariate analyses of our data. We therefore performed multiple-response testing, whereupon cardiovascular disease, hypertension, cancer, diabetes, and hyperlipidemia were all observed to contribute significantly to mortality among both unvaccinated and vaccinated individuals (Table 3). By contrast, obesity and respiratory illness were not found to be significant contributors, in contrast to previous reports [32,91,92]. Thyroid disease was likewise suggestive of inferior outcomes in prior studies [91,93], but was not found to be significant in this study. Figure 6 depicts increasing risk in individuals with greater numbers of comorbidities, similar to an earlier report which utilized the Charlson Comorbidity Index Score (CCIS) to address similar questions [94].

The relationship between specific SARS-CoV-2 variants and serious clinical outcomes was also examined, by leveraging the results of variant screening and sequencing data. Figure 7 suggests a slight increase in the frequency of severe outcomes as ancestral SARS-CoV-2 lineages were supplanted by earlier variants such as Iota (B.1.526), a former VOI which circulated in the New York/New Jersey region in early 2021 [95]. A decrease in severity was observed with Alpha (B.1.1.7), the first official VOC reported in late December 2020 [96], described as exhibiting increased receptor-binding affinity due to the N501Y spike mutation. This apparent decrease in clinical severity in our data may be related to increased vaccine uptake during the same time period, a trend which appears to have continued in mid-2021, when Delta (B.1.617.2) was the sole variant circulating.

A sharp increase in all three outcomes was subsequently observed from December 2021 to January 2022, following the emergence of the first Omicron sub-variant (BA.1), which was characterized by considerably more spike gene mutations than any preceding SARS-CoV-2 variant [97,98,99]. Despite perceived individual reduction in clinical severity from Omicron-related infection [26,29], the unprecedented transmissibility and infectivity resulted in large spikes in hospitalization, ICU admission, and mortality. Subsequent Omicron variants have exhibited much lower frequency of severe outcomes, likely due to widespread increase in herd immunity from both vaccination and natural infection. Notably, whereas a recent paper did not observe any differences in clinical outcome between the BA.1 and BA.2 waves [26], our data clearly shows a decrease in clinical severity during the BA.2 wave. This is likely because our study period extends through June 2022, allowing us to sample most of the first BA.2 wave.

Our study has several limitations worth mentioning, including the following. Sampling bias was present throughout the study period, as larger hospitals were likely over-represented, while smaller hospitals did not contribute representative numbers of swabs on a consistent basis. It is also possible that vaccine breakthrough swabs may have been over-sampled in our data set, thereby potentially skewing risk estimates. Our study also lacked a vaccinated control group with which to directly compare the efficacy of the three vaccine brands; nevertheless, Pfizer vaccine seemed to perform slightly better than Moderna or Janssen. We did not look at overall hospitalization rates, and chose to focus on severe clinical outcomes instead, which may have skewed our dataset towards patients with greater numbers of comorbidities. Moreover, our sample selection was limited to successfully screened swabs with lower Ct-values, potentially associated with higher viral load (although we did not observe differences in Ct-values for clinical outcomes). Our overall mortality rate (8.6%) was higher than that reported in similar studies [26], but comparable to that in a large recent national study [49]. It is possible that our mortality estimates include “all-cause mortality” in addition to COVID-related mortality. Lastly, risk factor data were not available for all subjects, and information regarding the temporal nature of comorbidities was lacking; therefore, inferences regarding the contribution of medical history to COVID-19 disease-related outcomes should be interpreted cautiously.

## 5. Conclusions

In conclusion, this study represents a thorough analysis of genomic, clinical, and epidemiologic factors among both vaccinated and unvaccinated individuals, during an eighteen-month period of the COVID-19 pandemic at a large healthcare organization in one of the most affected regions of the United States. We demonstrate the utility of rapid molecular diagnostic tools for variant surveillance, and their application to questions of clinical relevance, including vaccine efficacy. As next-generation COVID-19 vaccines continue to be developed [100], studies like ours will provide critical information regarding efficacy against diverse phylogenetic backgrounds [16,101,102]. The avenues of investigation described here offer continuing relevance for the ongoing SARS-CoV-2 pandemic, as well as future outbreaks of rapidly evolving viral pathogens.

## 6. Patents

Two of the authors (YZ and DSP) hold the following patents in relation to this study: “CDI Enhanced COVID-19 Test” (CA3169883A1) [8], and “CDI Rapid Test for COVID-19 Variants of Concern” (WO2022216821A1) [9].

## Figures and Tables

**Table 1:** Spike protein mutations used to detect specific SARS-CoV-2 variants of concern.

Variant of Concern	Pango Lineage	Spike Protein Mutations
**Alpha**	B.1.1.7	ΔH69/V70, N501Y
**Beta ^a^**	B.1.351	E484K, N501Y
**Gamma ^a^**	P.1 (B.1.1.28.1)	E484K, N501Y
**Delta**	B.1.617.2	L452R, T478K
**Omicron**	B.1.1.529	T478K, E484A, N501Y, Y505H
BA.1	B.1.1.529.1	A67V, ΔH69/V70, T478K, E484A, N501Y, Y505H
BA.2	B.1.1.529.2	T478K, E484A, N501Y, Y505H
BA.2.12.1	B.1.1.529.2.12.1	L452Q, T478K, E484A, N501Y, Y505H
BA.4/BA.5 ^b^	B.1.1.529.4/5	ΔH69/V70, L452R, T478K, E484A, F486V, N501Y, Y505H

^a^ Beta and Gamma could not be discriminated between without additional targets or WGS. ^b^ Omicron BA.4 and BA.5 could not be discriminated without additional targets or WGS.

**Table 2:** Severe clinical outcomes (ICU admission, ventilator support, and mortality) resulting from SARS-CoV-2 infection among vaccinated and unvaccinated individuals, stratified by age, gender, and race/ethnicity. Crude relative risks were calculated for particular outcomes by vaccination status, while adjusted relative risks were used to control for age, race/ethnicity and gender. RR, relative risk; CI, confidence interval; ICU, intensive care unit; IQR, interquartile range.

Outcome	Total	Unvaccinated	Vaccinated	RR_CRUDE_	95% CI	*p*-Value	RR_ADJUSTED_	95% CI	*p*-Value
**ICU Admissions, *n* (%)**	336 (6.7)	268 (7.8)	68 (4.3)	2.07	[1.51, 2.83]	<0.001			
Age, median (IQR)	68 (57–78)	67 (56–76)	72 (60–83)	1.07	[1.06, 1.08]	<0.001	1.07	[1.06, 1.09]	<0.001
Gender, *n* (%)									
Male	197 (58.6)	154 (57.5)	43 (63.2)	1.82	[1.41, 2.36]	<0.001	0.79	[0.57, 1.09]	0.16
Female	139 (41.4)	114 (42.5)	25 (36.8)	0.55	[0.42, 0.71]	<0.001	1.26	[0.91, 1.75]	0.16
Race/Ethnicity, *n* (%)									
Hispanic	59 (17.6)	49 (18.3)	10 (14.7)	0.95	[0.51, 1.74]	0.87	0.52	[0.20, 1.26]	0.15
Asian	15 (4.5)	12 (4.5)	3 (4.4)	2.49	[0.37, 1.70]	0.08	1.05	[0.29, 4.56]	0.94
Black	22 (6.5)	21 (7.8)	1 (1.5)	0.35	[0.14, 0.79]	0.02	2.84	[0.61, 41.66]	0.28
White	210 (62.5)	161 (60.1)	49 (72.1)	1.48	[0.94, 2.36]	0.09	0.72	[0.32, 1.36]	0.35
**Ventilator Support, *n* (%)**	236 (4.7)	185 (5.4)	51 (3.2)	1.85	[1.30, 2.69]	<0.001			
Age, median (IQR)	70 (60–79)	69 (60–78)	72 (64–83)	1.09	[1.07, 1.10]	<0.001	1.09	[1.07, 1.10]	<0.001
Gender, *n* (%)									
Male	141 (59.7)	109 (58.9)	32 (62.7)	1.90	[1.40, 2.56]	<0.001	0.83	[0.57, 1.21]	0.34
Female	95 (40.3)	76 (41.1)	19 (37.3)	0.53	[0.39, 0.71]	<0.001	1.20	[0.83, 1.76]	0.34
Race/Ethnicity, *n* (%)									
Hispanic	49 (20.8)	39 (21.1)	10 (19.6)	1.31	[0.66, 2.57]	0.44	1.04	[0.48, 2.21]	0.91
Asian	10 (4.2)	8 (4.3)	2 (3.9)	1.89	[0.50, 5.90]	0.31	2.23	[0.49, 7.93]	0.25
Black	17 (7.2)	17 (9.2)	0 (0.0)	0.41	[0.15, 1.03]	0.07	0.56	[0.20, 1.46]	0.26
White	137 (58.1)	104 (56.2)	33 (64.7)	1.89	[0.64, 1.89]	0.75	1.06	[0.58, 1.96]	0.86
**Mortality, *n* (%)**	430 (8.6)	320 (9.3)	110 (7.0)	1.44	[1.11, 1.87]	0.01			
Age, median (IQR)	75 (65–85)	74 (63–85)	79 (68–87)	1.14	[1.13, 1.16]	<0.001	1.14	[1.13, 1.16]	<0.001
Gender, *n* (%)									
Male	245 (57.0)	174 (54.4)	71 (64.5)	2.03	[1.56, 2.66]	<0.001	0.7	[0.53, 0.92]	0.01
Female	185 (43.0)	146 (45.6)	39 (35.5)	0.59	[0.46, 0.74]	<0.001	1.43	[1.09, 1.87]	0.01
Race/Ethnicity, *n* (%)									
Hispanic	58 (13.5)	44 (13.8)	14 (12.7)	1.23	[0.57, 2.65]	0.60	0.46	[0.21, 0.99]	0.05
Asian	16 (3.7)	13 (4.1)	3 (2.7)	1.76	[0.42, 5.63]	0.38	1.76	[0.54, 7.40]	0.38
Black	25 (5.8)	23 (7.2)	2 (1.8)	0.22	[0.04, 0.74]	0.03	2.14	[0.63, 12.07]	0.29
White	304 (70.7)	220 (68.8)	84 (76.4)	3.33	[1.94, 6.15]	<0.001	0.84	[0.46, 1.44]	0.55

**Table 3:** Results of multiple-response testing using a Poisson distribution, highlighting significant contributions to mortality among individual risk factors, ranked in descending order. The length of the significance bar depicts the strength of the association.

Comorbidity	Chi-Square Statistic	Significance Bar	*p*-Value
Heart Disease	44.54	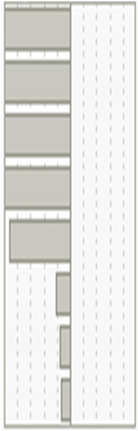	<0.001
Hypertension	41.06		<0.001
Cancer	34.45		<0.001
Diabetes	31.02		<0.001
Hyperlipidemia	17.61		<0.001
Obesity	3.1		0.10
Thyroid Disease	1.82		0.20
Respiratory Disease	1.47		0.23

## Data Availability

The SARS-CoV-2 genomes sequenced in this study were deposited in GISAID (https://www.gisaid.org). Sequences can be accessed by searching records from both the originating lab at Hackensack Medical Center and the submitting labs at the New York Genome Center and the New Jersey Public Health and Environmental Laboratories.

## Supplementary Materials

The following supporting information can be downloaded at: https://www.mdpi.com/article/10.3390/v15081699/s1, Figure S1: COVID-19 Hospital Census Data, 1 March 2020–30 June 2023; Figure S2: Severe COVID-19 Hospitalization Outcomes, 1 March 2020–30 June 2023; Table S1: Demographics of De-Identified Subjects Positive for SARS-CoV-2; Table S2: Severe Clinical Outcomes Observed Among SARS-CoV-2 Variants.
