# Supplementary material for: Molecular and Clinical Epidemiology of SARS-CoV-2 Infection among Vaccinated and Unvaccinated Individuals in a Large Healthcare Organization from New Jersey
Source: Viruses. 2023 Aug 5;15(8):1699. doi: 10.3390/v15081699 (PMC10457875; doi:10.3390/v15081699)
Supplement: Supplementary file 1 [file viruses-15-01699-s001.zip › viruses-2529469-supplementary.pdf]

SUPPLEMENTAL DATA:

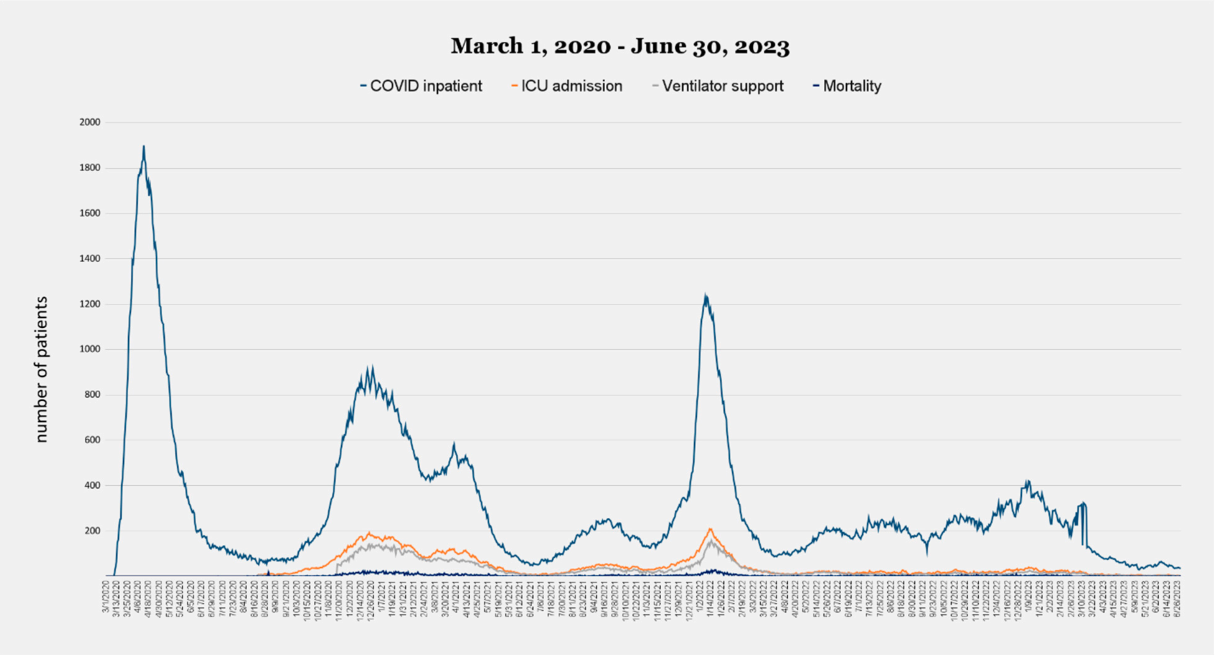

Figure S1. COVID-19 Hospital Census Data, March 1, 2020 – June 30, 2023. Longitudinal chart depicting hospitalized patients in 17 network hospitals since March 1, 2020. Dark blue line represents the daily census of COVID-related inpatients; orange line represents daily census of COVID patients in intensive care (data available from August 2020); gray line represents daily census of COVID patients on ventilator support (data available from November 2020); black line represents daily census of COVID inpatient deaths (data available from August 2020). Three prominent surge periods are discernible, corresponding to: a) the initial onset of the pandemic (Mar - Jun 2020); b) the first winter surge (Nov 2020 - Feb 2021); and c) the second winter surge, driven largely by the emergence of the Omicron variant (Dec 2021 - Feb 2022). Data retrieved from Hackensack Meridian Health as of June 30, 2023.

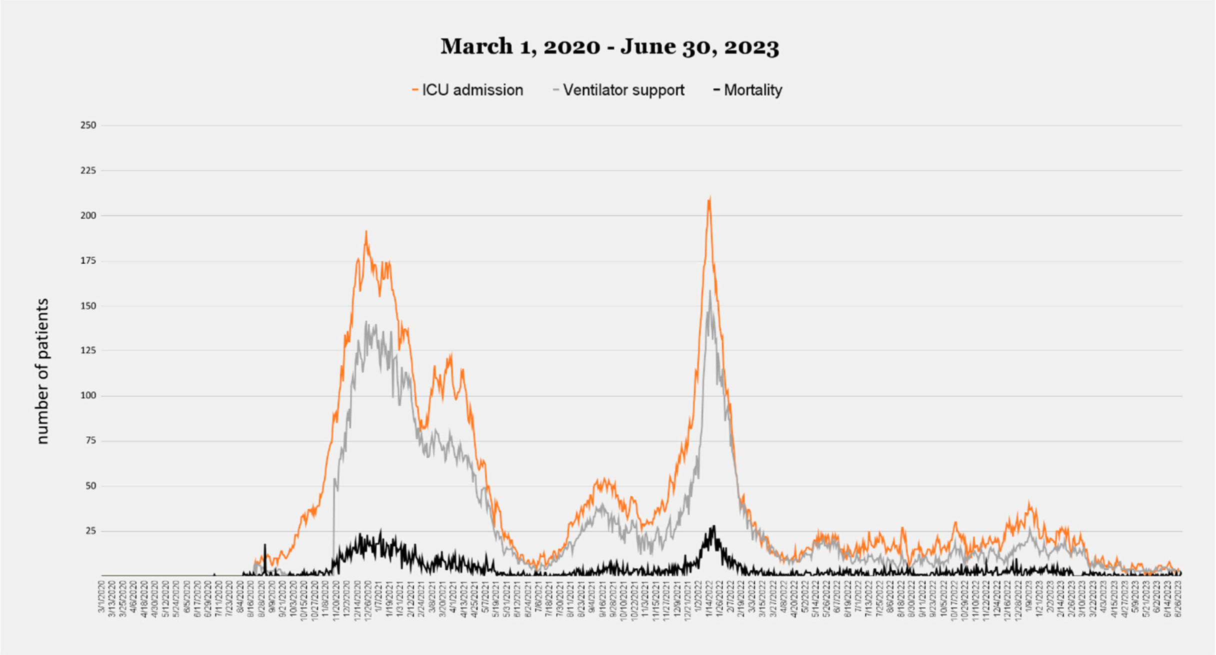

Figure S2. Severe COVID-19 Hospitalization Outcomes, March 1, 2020 – June 30, 2023. Same as Figure S1, only without COVID inpatient data, in order to highlight ICU admissions (orange line), ventilator support (gray line), and mortality (black line). The number of patients for all three outcomes was higher during the first Omicron surge (Dec 2021 - Feb 2022) than during the first winter surge (Nov 2020 - Feb 2021) or the Delta period (May 2021 – November 2021). Data retrieved from Hackensack Meridian Health as of June 30, 2023.

Demographics	Unvaccinated (*N*=3,430) *n* (%)	Vaccinated (*N*=1,577) *n* (%)	*p*-value	Total (*N*=5,007) *n* (%)
**Age Group**				
0 - 4	245 (7.1)	0 (0)	*	245 (4.9)
5 - 17	253 (7.4)	31 (2.0)	**<0.001**	284 (5.7)
18 - 29	353 (10.3)	119 (7.5)	**<0.001**	472 (9.4)
30 - 39	427 (12.4)	170 (10.8)	0.16	597 (11.9)
40 - 49	376 (11.0)	159 (10.1)	0.48	535 (10.7)
50 - 59	450 (13.1)	220 (14.0)	0.32	670 (13.4)
60 - 69	511 (14.9)	266 (16.9)	0.06	777 (15.5)
70 - 79	423 (12.3)	306 (19.4)	**<0.001**	729 (14.6)
80 - 89	293 (8.5)	230 (14.6)	**<0.001**	523 (10.4)
> 89	99 (2.9)	76 (4.8)	**<0.001**	175 (3.5)
**Gender**				
Male	1627 (47.4)	687 (43.6)	**0.01**	2314 (46.2)
Female	1803 (52.6)	890 (56.4)	-	2693 (53.8)
**Race / Ethnicity**				
White (non-Hispanic)	1867 (54.4)	1039 (65.9)	**<0.001**	2906 (58.0)
Black (non-Hispanic)	396 (11.5)	113 (7.2)	**<0.001**	509 (10.2)
Asian (non-Hispanic)	106 (3.1)	65 (4.1)	**<0.001**	171 (3.4)
Other (non-Hispanic)	357 (10.4)	157 (10.0)	0.51	514 (10.3)
Hispanic	704 (20.5)	203 (12.9)	**<0.001**	907 (18.1)
**Variant / Subvariant**				
**Alpha**	688 (20.1)	26 (1.6)	**<0.001**	714 (14.3)
**Delta**				
B.1.617.2	122 (3.6)	65 (4.1)	0.35	187 (3.7)
Delta sub-variants	1159 (33.8)	531 (33.7)	-	1690 (33.8)
**Omicron**				
BA.1	462 (13.5)	437 (27.7)	0.31	899 (18.0)
BA.2	434 (12.7)	419 (26.6)	0.23	853 (17.0)
BA.4 / BA.5	30 (0.9)	20 (1.3)	0.22	50 (1.0)

**Other**				
Beta (B.1.351)	1 (<0.1)	0 (0.0)	*	1 (<0.1)
Epsilon (B.1.429)	7 (0.2)	2 (0.1)	*	9 (0.2)
Gamma (P.1)	37 (1.1)	2 (0.1)	0.25	39 (0.8)
Iota (B.1.526)	219 (6.4)	15 (1.0)	0.28	234 (4.7)
Kappa (B.1.617.1)	1 (<0.1)	0 (0.0)	*	1 (<0.1)
Lambda (C.37)	1 (<0.1)	0 (0.0)	*	1 (<0.1)
Mu (B.1.621)	14 (0.4)	1 (0.1)	*	15 (0.3)
Zeta (P.2)	1 (<0.1)	0 (0.0)	*	1 (<0.1)
**Early Lineages**	254 (7.4)	59 (3.7)	**<0.001**	313 (6.3)

Table S1. Demographic characteristics of de-identified subjects represented in this study, stratified by COVID-19 vaccination status and SARS-CoV-2 variant infection. "Early Lineages" corresponds to pre-variant lineages described in the legend for Figure 2. No children under the age of 5 were vaccinated during the timeframe encompassed by this study. Significant *p*-values (< 0.05) are shown in boldface. Significance testing was not performed when sample sizes were too low (denoted by *).

**Outcome**	**Total**	**Unvaccinated**	**Vaccinated**	**RR_{Crude}**	**95% CI**	***p*-value**	**$RR_{Adjusted}$**	**95% CI**	***p*-value**
**ICU Admissions *vs.* SARS-CoV-2 Variant, *n* (%)**									
Early Lineages	18 (5.9)	16 (6.5)	2 (3.5)	2.17	[0.49, 18.48]	0.38	1.55	[0.34,13.39]	0.62
Other	29 (9.3)	43 (8.0)	4 (5.1)	0.97	[0.22, 8.36]	0.97	1.24	[0.26, 10.95]	0.81
Alpha	62 (8.9)	60 (8.7)	2 (7.7)	1.17	[0.28, 9.77]	0.86	1.37	[0.32, 11.56]	0.72
Delta	104 (5.5)	81 (6.3)	23 (3.9)	1.82	[1.08, 3.22]	**0.03**	3.01	[1.74, 5.44]	**<.001**
Omicron BA.1	87 (9.7)	64 (13.9)	23 (5.3)	3.40	[1.95, 6.15]	**<.001**	4.83	[2.73, 8.85]	**<.001**
Omicron BA.2	34 (4.0)	18 (4.2)	16 (3.8)	1.10	[0.50, 2.46]	0.81	2.06	[0.92, 4.66]	0.08
Omicron BA.4/BA.5	2 (4.0)	2 (6.7)	0 (0.0)	*	*	*	*	*	*
**Ventilator Support *vs.* SARS-CoV-2 Variant, *n* (%)**									
Early Lineages	12 (4.0)	11 (4.5)	1 (1.7)	3.11	[0.46, 89.24]	0.35	2.12	[0.30, 61.32]	0.54
Other	21 (6.8)	19 (6.6)	2 (9.5)	0.63	[0.13, 5.47]	0.60	0.82	[0.16, 7.47]	0.83
Alpha	40 (5.6)	38 (5.5)	2 (7.7)	0.66	[0.15, 5.60]	0.64	0.69	[0.15, 5.98]	0.68
Delta	82 (4.4)	60 (4.7)	22 (3.7)	1.33	[0.76, 2.42]	0.33	2.20	[1.22, 4.10]	**0.01**
Omicron BA.1	56 (6.2)	45 (9.7)	11 (2.5)	5.19	[2.49, 11.92]	**<.001**	7.68	[3.63, 17.87]	**<.001**
Omicron BA.2	24 (2.8)	11 (2.5)	13 (3.1)	0.79	[0.30, 2.01]	0.62	1.54	[0.58, 4.01]	0.37
Omicron BA.4/BA.5	1 (2.0)	1 (3.3)	0 (0.0)	*	*	*	*	*	*
**Mortality *vs.* SARS-CoV-2 Variant, *n* (%)**									
Early Lineages	36 (11.9)	34 (13.9)	2 (3.5)	5.67	[1.37, 46.96]	**0.04**	3.33	[0.76, 28.37]	0.17
Other	39 (12.5)	37 (12.8)	2 (9.5)	1.46	[0.33, 12.45]	0.67	2.26	[0.46, 20.36]	0.38
Alpha	74 (10.4)	70 (10.2)	4 (15.4)	0.58	[0.17, 2.45]	0.40	0.45	[0.18, 1.39]	0.12
Delta	164 (8.7)	108 (8.4)	56 (9.4)	0.87	[0.59. 1.30]	0.49	0.68	[0.39, 1.14]	0.16
Omicron BA.1	89 (9.9)	57 (12.3)	32 (7.3)	1.94	[1.16, 3.32]	**0.01**	3.38	[1.95, 5.96]	**<.001**
Omicron BA.2	27 (3.2)	14 (3.2)	13 (3.1)	1.05	[0.43, 2.57]	0.92	2.35	[0.94, 5.91]	0.07
Omicron BA.4/BA.5	1 (2.0)	0 (0.0)	1 (5.0)	*	*	*	*	*	*

Table S2. Severe clinical outcomes (ICU admission, ventilator usage, and mortality) resulting from COVID-19 infection among vaccinated and unvaccinated individuals, stratified by SARS-CoV-2 variant. Crude relative risk refers to the overall risk of a particular outcome among the total number of individuals in a particular category, while adjusted relative risk refers to the risk after adjusting for age, gender and race/ethnicity. Significant *p*-values (< 0.05) are shown in boldface. RR. relative risk; CI, confidence interval; ICU, intensive care unit.
